# Enhancing Recovery and Performance Through Plyometric Training for a Badminton Athlete After Anterior Cruciate Ligament (ACL) Reconstruction: A Case Report

**DOI:** 10.7759/cureus.67636

**Published:** 2024-08-23

**Authors:** Saylee S Shedge, Swapnil U Ramteke

**Affiliations:** 1 Sports Physiotherapy, Ravi Nair Physiotherapy College, Datta Meghe Institute of Higher Education and Research, Wardha, IND

**Keywords:** explosive power, athlete, stretch shortening cycle, sports rehabilitation, plyometric training, badminton, acl reconstruction

## Abstract

Anterior cruciate ligament (ACL) injuries are widespread, particularly in sports that involve rapid changes in direction, such as badminton, and it incapacitates an athlete severely and for a long time. ACL reconstruction followed by a structured rehabilitation program is essential for returning to peak performance. Plyometric training, known for enhancing explosive power and agility, is increasingly incorporated in post-ACL reconstruction rehabilitation for athletes. This case report presents the rehabilitation of a 19-year-old female badminton player operated for ACL reconstruction through the inculcation of plyometric training in the later phase to optimize better performance outcomes. The athlete demonstrated significant improvements in knee stability, strength, and functional performance. Plyometric exercises played a crucial role in restoring explosive power and agility, essential for competitive badminton, thereby facilitating a successful return to sport.

## Introduction

In the world of recreational and competitive sports, badminton is among the most widely embraced racket sport by people for leisure and professional contests. Thus, badminton is played by young and old, professionals, and amateurs. Adequate body position sense, rapid hand-eye coordination, rapid arm movements, quick change of direction (COD), lunges, and frequent jumps are required in badminton [1]. Many anterior cruciate ligament (ACL) injuries happen in sports during noncontact direction changes, such as cutting, pivoting, and plant-and-cut actions. This is because these movements produce high forces and load the knee joint in multiple planes (sagittal, frontal, and transverse) when the foot plants to change direction, leading to increased strain on the ACL [2]. Anterior cruciate ligament injuries happen when the stress placed on the ligament surpasses its tolerance limit [2]. Badminton players need excellent active stability to perform speedy movements on the court effectively and avoid injuries [3].

Plyometric training minimizes the firing rate of sensitive Golgi tendon organs to excessive muscle tension allowing for the muscle’s stretchy elements to elongate [4]. According to Lephart et al., reducing Golgi tendon organ sensitivity enhances the function of muscle spindles, thereby improving proprioception [5]. Plyometric training boosts reflex potentiation and alters the elastic properties of muscles and connective tissue by improving the neural recruitment of motor units or increasing neural firing frequency, thereby enhancing neuromuscular adaptability [6]. Some physiological adaptations to plyometric programs include improving muscular power and strength, enhancing functional patterns for dynamic joint stability, quicker muscle response, and preventing further injuries [7]. Plyometrics has been proven effective not only in sports but also in daily functional life, but there is less evidence of the same in terms of power and agility [8]. Prior studies have indicated that plyometric training is useful in improving leg extensor power, strength, and high potential dynamic movements significantly, accentuating the vertical jumping ability [9]. The plyometric program develops explosiveness, that is, to use strength as quickly and vigorously as possible, and this covers the gap between strength and velocity so the athlete can maximize power production [10].

The purpose of this case report is to demonstrate the effectiveness of plyometric training in optimizing the performance and recovery of a badminton athlete who has undergone ACL reconstruction. It aims to highlight the specific benefits of integrating plyometric exercises into rehabilitation protocols. Additionally, the report seeks to provide evidence-based insights for enhancing athletic performance post surgery.

## Case presentation

Patient information

A 19-year-old female badminton player attended a badminton coaching camp in July last year. On the first day of the camp, during a high-intensity rally, she lunged forward to execute a powerful forehand smash. This movement caused a sudden pivot change in direction, resulting in her falling to the ground. She reported hearing a click sound from her right knee and immediately experienced pain at the back of the knee, which gradually increased as she attempted to get up and move her leg. Progressive swelling developed over the knee joint, for which she applied ice and an ointment for pain. The swelling remained constant as she did not continue with icing. After the camp was over, on the next day, she visited a government hospital, where the doctor performed an anterior drawer test that suggested a possible ACL tear. She was recommended to wear a long knee brace and was advised to go for a magnetic resonance imaging (MRI). A week later, the patient underwent an MRI at a local hospital in Nagpur. The MRI report received 15 days later revealed a complete-thickness ACL tear with mild knee joint effusion. The patient sought a second opinion for which she consulted a local physician in Hinganghat. The doctor confirmed an ACL tear based on the MRI report and advised her to take rest. The patient took a rest at home for two months without taking any medications. Since the pain was not subsiding, she was then referred to the Acharya Vinoba Bhave Rural Hospital (AVBRH) for further management. In September, she visited the AVBRH orthopedic outpatient department (OPD) and was advised to get admitted for ACL reconstruction surgery. After admission, she was prescribed painkillers, calcium, and vitamin supplements. The patient underwent arthroscopic ACL reconstruction. The graft was taken from the peroneus longus muscle. She was discharged two weeks postoperatively. Following the surgery, she immediately began physiotherapy rehabilitation.

Clinical findings

The patient visited the sports outpatient department with complaints of pain in the right knee and difficulty in completely flexing the knee joint. She also complained of impaired balance. The pain was of a dull aching nature. The pain was 5.3/10 on activity and 0/10 at rest on the visual analogue scale. A tenderness of grade 2 in the right knee was confirmed by palpation. The before and after intervention assessment of the affected and non-affected limb’s range of motion is elicited in Table 1 and muscle strength in Table 2. Therefore, an appropriate treatment regime was prescribed; the main aim of physical therapy was to alleviate the pain and help regain muscular strength. In the later phase, because she was a badminton player, plyometric training was also included.

**Table 1:** Range of motion of the tight (affected) and left lower limb

Joint	Movement	Right		Left
		Pre-treatment	Post-treatment	
Hip	Flexion	0-80°	0-120°	0-120°
	Extension	0-15°	0-30°	0-30°
	Abduction	0-30°	0-40°	0-40°
	Adduction	0-30°	0-30°	0-30°
Knee	Flexion	10-90°	0-140°	0-140°
	Extension	90-10°	140-0°	140-0°
Ankle	Dorsiflexion	0-15°	0-20°	0-20°
	Plantar flexion	0-40°	0-50°	0-50°
	Inversion	0-20°	0-30°	0-30°
	Eversion	0-20°	0-20°	0-20°

**Table 2:** Manual muscle testing of the right (affected) and left lower limb Grade 3, full ROM against gravity; grade 4, full ROM against gravity moderate resistance; and grade 5, full ROM against gravity maximum resistance ROM: range of motion

Joint	Muscles	Right		Left
		Pre-treatment	Post-treatment	
Hip	Flexors	3/5	5/5	5/5
	Extensors	3/5	5/5	5/5
	Abductors	3/5	5/5	5/5
	Adductors	4/5	5/5	5/5
Knee	Flexors	3/5	5/5	5/5
	Extensors	3/5	5/5	5/5
Ankle	Dorsiflexors	4/5	5/5	5/5
	Plantar flexors	4/5	5/5	5/5
	Invertors	4/5	5/5	5/5
	Evertors	4/5	5/5	5/5

Clinical investigations

**Figure 1:**
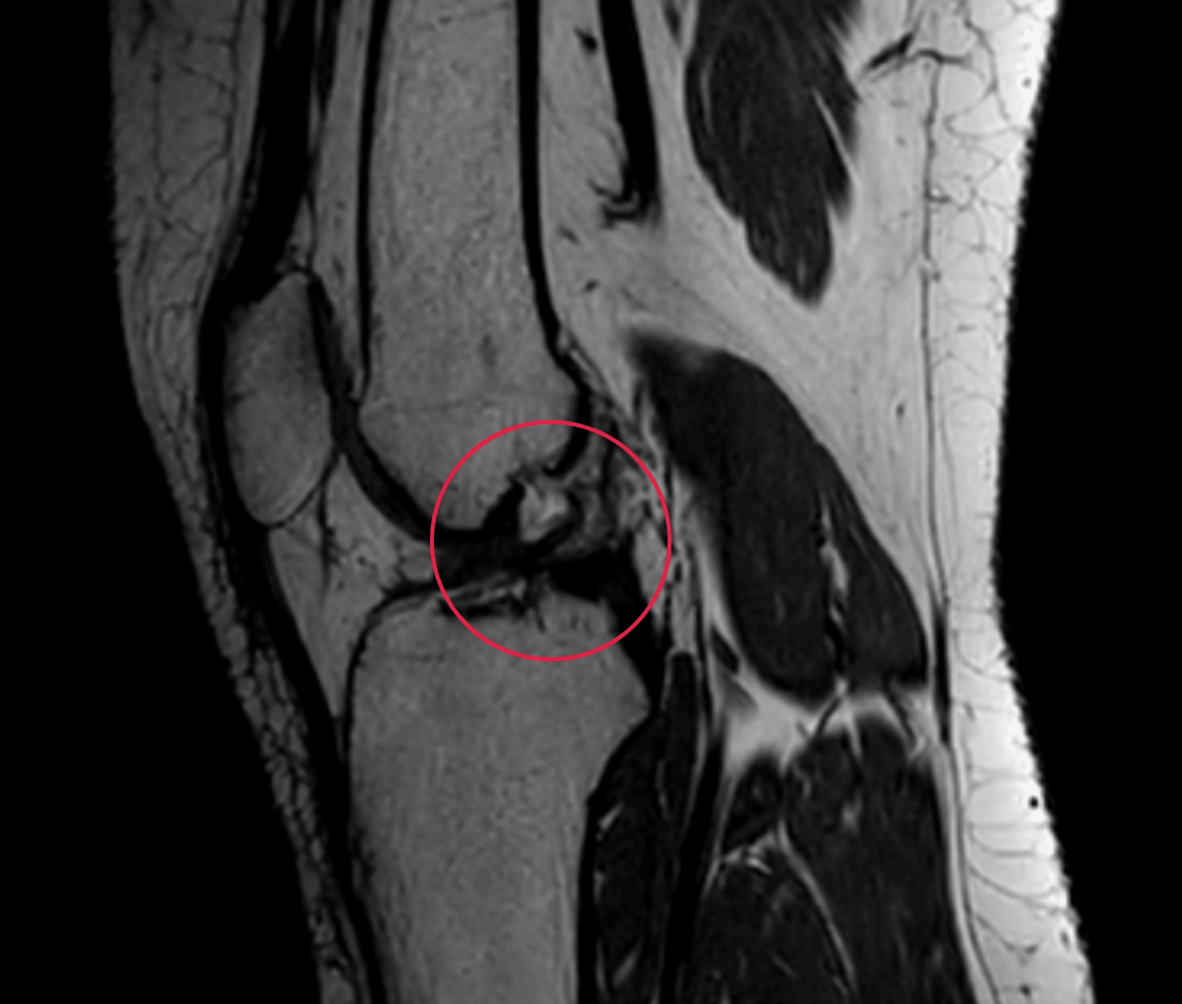
An MRI of the right knee showing a complete-thickness ACL tear The circle represents a complete-thickness ACL tear MRI, magnetic resonance imaging; ACL, anterior cruciate ligament

An MRI of the right knee was done to confirm the anterior cruciate ligament tear (Figure 1).

Outcome measures

The pre- and post-treatment outcome measures used were the visual analogue scale, lower quarter Y balance test, single hop test, and triple hop test, as elicited in Table 3.

**Table 3:** Outcome measures VAS: visual analogue scale

Serial number	Outcome measures		Pre-treatment	Post-treatment
1	VAS	On activity	5.3/10	1.2/10
		At rest	0/10	0/10
2	Lower quarter Y balance test		Left: 81.22%	Left: 93.85%
			Right (affected): 65.20%	Right (affected): 88.36%
3	Single hop test		Right (affected): 70 cm	Right (affected): 115 cm
			Left: 110 cm	Left: 122 cm
4	Triple hop test		Right (affected): 219 cm	Right (affected): 345 cm
			Left: 336 cm	Left: 352 cm

**Figure 2:**
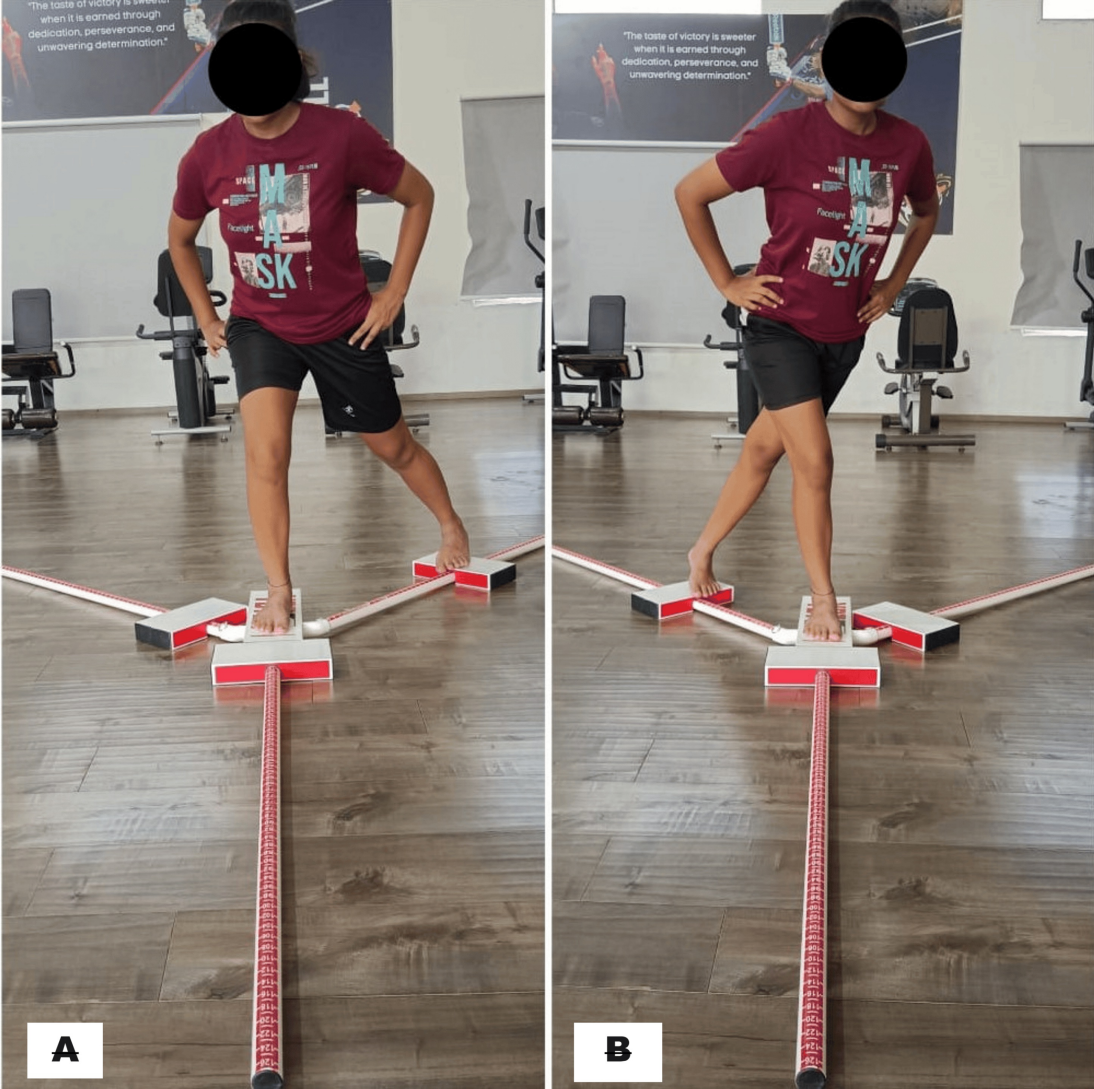
Lower quarter Y balance test (A) Right posteromedial reach and (B) right posterolateral reach

Figure 2 shows the dynamic balance assessment through the Y balance test.

Physiotherapy rehabilitation

In patient care, a physical therapy rehabilitation program, as shown in Table 4, and plyometric training in Table 5 were framed.

**Table 4:** Physiotherapy treatment protocol NMES, neuromuscular electrical stimulation; VMO, vastus medialis oblique; ROM, range of motion; SLR, straight leg raise

Serial number	Goal	Intervention	Rationale
1	To aid recovery	Advice to wear a long knee brace for 14-16 hours/day	To maximize the stability of the knee joint and to avoid excessive loading on the surrounding structures of the joint
	To maintain joint stability and mobility	Begin with isometric exercises twice a day for 10 repetitions/two sets for the major muscles of the knee such as the quadriceps and hamstrings	Alleviates joint effusion, and enhances blood flow to the joint, which contributes to faster healing and strengthens the muscles with minimal tension
2	To prevent the neurophysiological inhibition of quadriceps muscle	NMES for VMO muscle activation. Prone knee hangs with weight cuff and passive range of motion for full knee extension two times a day for two sets of 10 repetitions	Reeducates the muscle, and maintains joint integrity by inhibiting extensor lag to occur
3	To avoid stiffness and restricted ROM	Knee flexion ROM exercises thrice daily for 10 repetitions/three sets	Faster improvement to regain the range
4	To increase the strength of the muscles	Multiangle SLR in all planes, multiangle hip abductor strengthening, terminal knee extension on Swiss ball, fire hydrants, clamshell exercise, and active knee extension twice daily for three sets of 10 repetitions each	Rehabilitation of the muscles for quicker recovery
5	To readapt to daily life and functional movements	Minimal weight-bearing with the brace initially progressing to partial weight-bearing and then full weight-bearing.	Bedside mobility is enhanced, which in turn promotes earlier return to daily activities by ensuring that the graft and surrounding tissues heal properly while gradually restoring muscle strength, joint stability, and functional mobility

**Table 5:** Plyometric training protocol The rest period between exercises is 60 seconds and between sets is three minutes [11]

Exercises	Weeks 1 and 2	Weeks 3 and 4	Weeks 5 and 6
Front barrier jump	Ten repetitions × three sets	Five repetitions × three sets	Five repetitions × four sets
Lateral high knees with hurdles	Ten repetitions × three sets	Four repetitions × three sets	Six repetitions × three sets
Lateral barrier jump	Ten repetitions × three sets	Twelve repetitions × three sets	Fifteen repetitions × three sets
Multi-directional jumps with hurdles	Ten repetitions × three sets	Eight repetitions × three sets	Twelve repetitions × three sets

**Figure 3:**
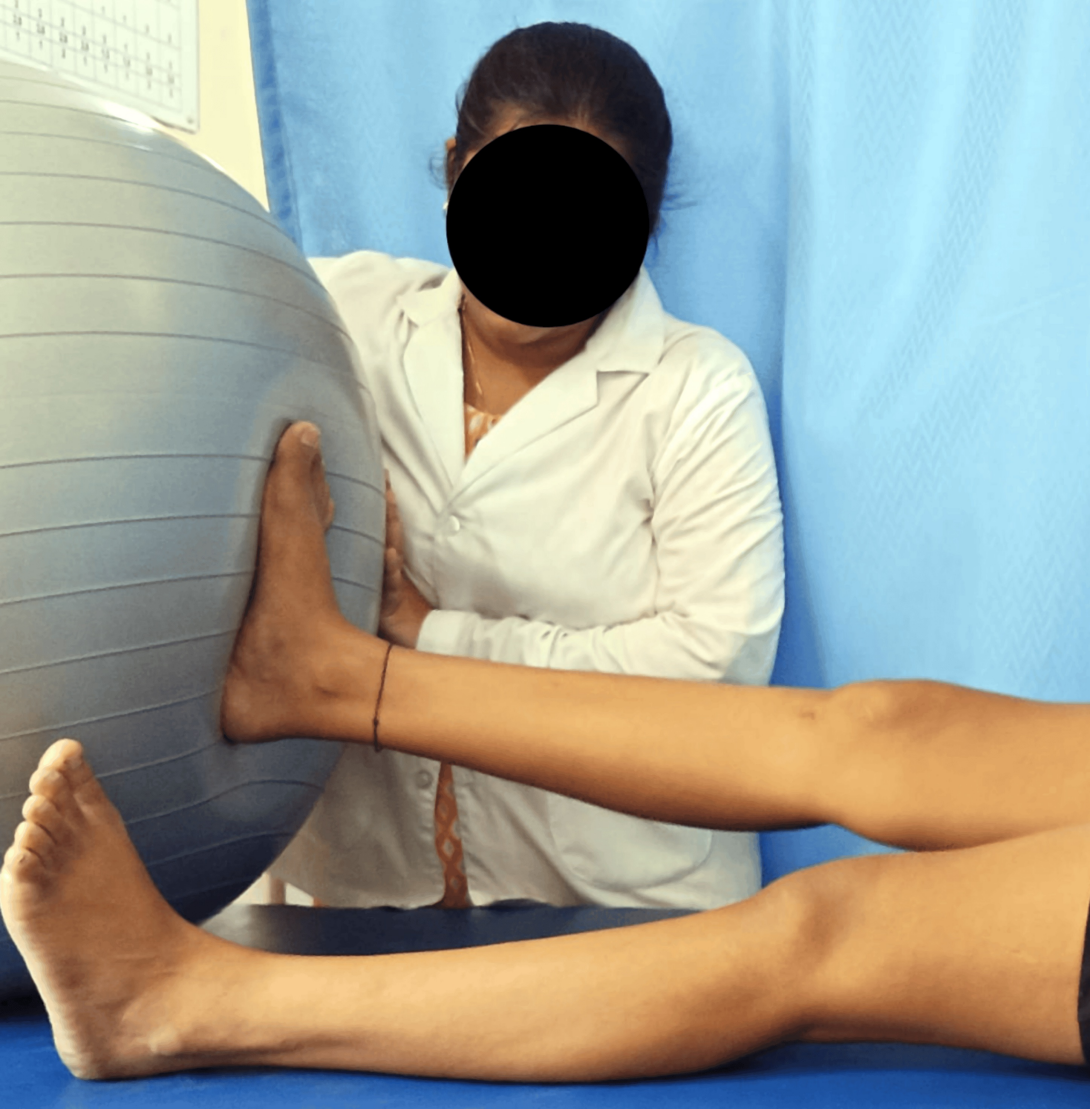
Terminal knee extension on Swiss ball

Terminal knee extension on Swiss ball exercise is shown in Figure 3.

**Figure 4:**
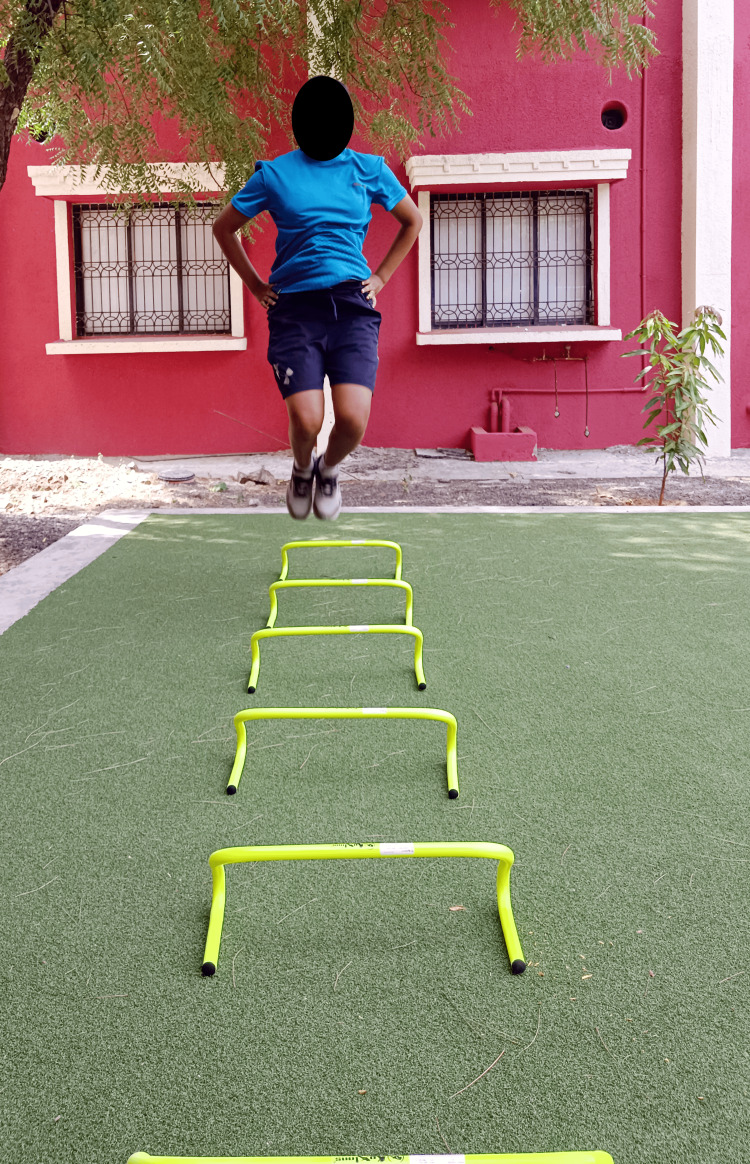
Front barrier jumps

**Figure 5:**
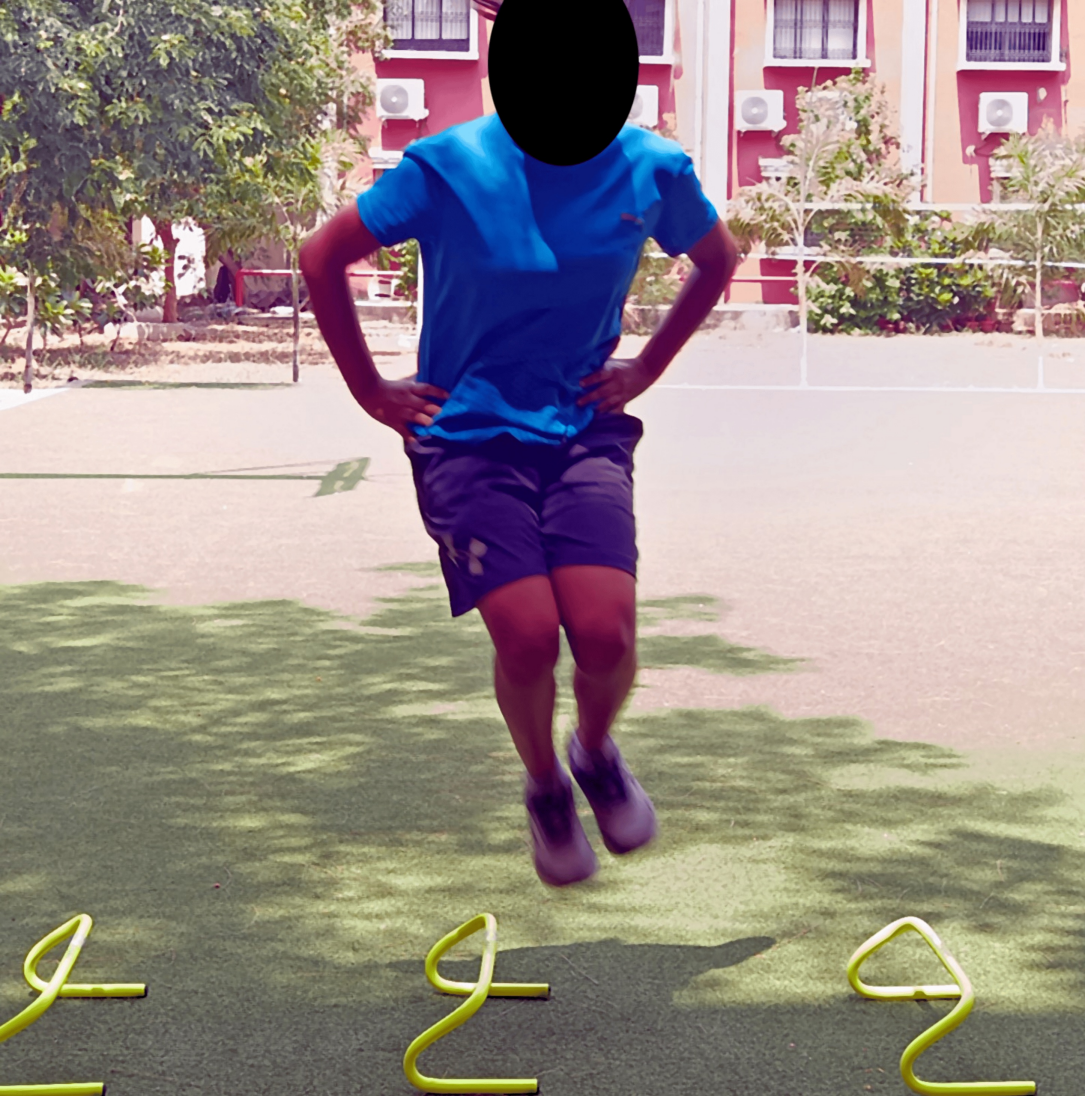
Lateral barrier jumps

**Figure 6:**
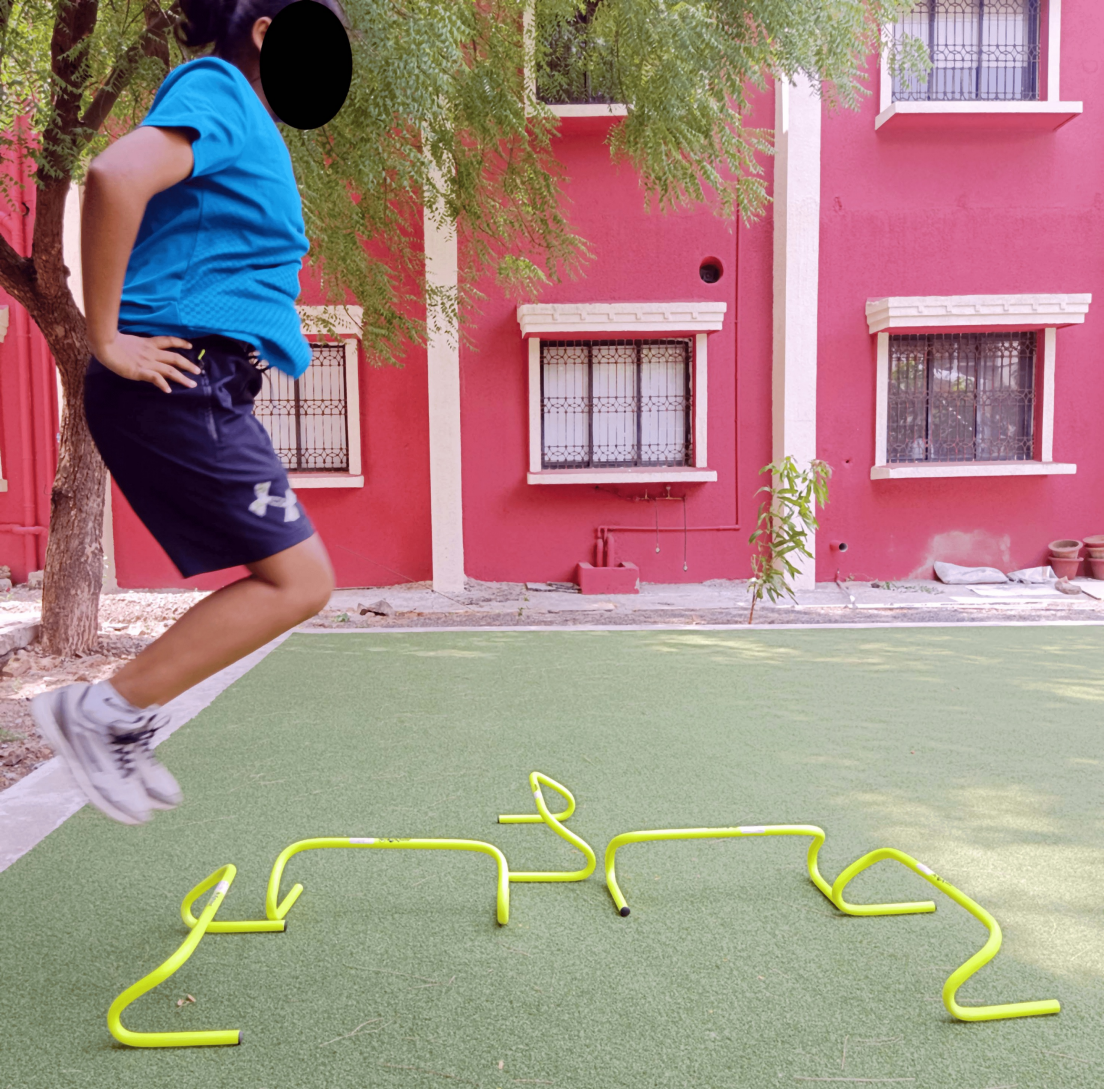
Multi-directional jumps

Figures 4-6 show plyometric exercises.

## Discussion

Anterior cruciate ligament (ACL) reconstruction is generally performed on athletes with ACL tear; full reconstruction is achieved, but the best management of the affected limb is still a topic of active research. The training termed plyometrics, which has been proven to increase muscular power and agility, has been effective in increasing the recovery and performance of sportsmen. This present case report aims to describe the integration of plyometric training in the rehabilitation process of a badminton player with an ACL tear managed through reconstruction. With regard to the movement patterns, hence enabling a more effective return to play, this approach is characterized by the performance of movements specific to the sporting discipline and high-intensity training. The study informs the understanding of the efficacy of plyometrics in the rehabilitation program of a badminton athlete under the post-ACL reconstruction program.

The experimental study by Chandra et al. involved 102 participants divided into the experimental group and control group (CG) randomly. Plyometric training was done by the experimental group for three weeks, while the control group carried on with their regular training session but excluded the plyometric training. The t-test for agility, the 30 m sprint for speed, and the broad jump test for aerobic fitness were used as indicators for this study’s outcome measures. Before and after the intervention, the related results showed a difference in agility, speed, and explosive power in the experimental group, hence concluding that plyometric training was more effective than the regular training regime [12].

Sinkovic et al. performed a randomized controlled trial on young tennis players, which included 35 male tennis players, who were randomly recruited to the control group where they carried out their regular conditioning training with standard technical-tactical training, and the experimental group performed plyometric exercises in their additional technical-tactical training for six weeks. The subjects had to undergo a series of tests, which consisted of 5, 10, and 20 m sprints, 20 yd run, 4 × 10 yd run, t-test, tennis-specific change of direction speed (TENCODS) test, and tennis-specific reactive agility (TENRAG) test. The study concluded that the experimental group showed significant betterment in the variables after the intervention protocol when the final testing was done [13].

Thotawaththa et al. executed a study on elite squash players, which consisted of 11 elite squash players assigned to an eight-week plyometric program as an addition to their regular squash practice. The assessment measures included the 505 agility test, vertical jump test, 10 m sprint test, and change of direction (COD) deficit calculator. The study showed a piece of powerful evidence supporting the benefits of plyometric training for improvement in the change of direction speed, agility, acceleration, and power [14].

The semi-experimental study by Alikhani et al. was conducted on female badminton players. Twenty-two physically active badminton players participated in the study and were selected randomly for the control and experimental groups where the latter was to undergo plyometric training for six weeks. Y balance test for the assessment of dynamic balance and photography method for the assessment of knee proprioception were performed before and after the training intervention. The experimental group demonstrated a better outcome in the dynamic balance and knee proprioception of the badminton players [1].

In a study carried out by Ozmen and Aydogmus on 20 adolescent badminton players randomly divided into two groups, the plyometric group (PG) and the control group (CG). The outcomes used in the study were the Illinois agility test and the vertical squat jump test. The findings observed that there was an improvement in squat jump in the PG over the CG due to neural factors because the training duration used was less than eight weeks. The study also revealed an improvement in agility time since plyometric exercises contain lower extremity explosive movements and fast muscle contraction as it implies the ability to control the body position and the ability to change directions promptly, defined as agility. Therefore, the PG was more superior than the CG [15].

In the study done on a total of 42 students, Lim et al. randomly assigned the participants to the control group and the experimental group. Both the groups executed the co-curriculum program concurrently with the experimental group involved in the plyometric program additionally. The outcome measure used was the Illinois agility test. The study concluded that there was an increase in the agility scores of both the control and experimental groups, but the change in the experimental group was higher than that of the control group [16].

## Conclusions

The incorporation of plyometric training into the rehabilitation program of badminton athletes post ACL reconstruction demonstrated notable improvements in enhancing both recovery and athletic performance. Plyometric exercises played a crucial role in accelerating the rehabilitation process and minimizing the risk of reinjury. This targeted approach facilitated a quicker return to competitive play by improving muscle strength, joint stability, agility, and explosive power. The athlete not only regained endurance but also demonstrated improved on-court performance, underscoring the value of a well-structured, sport-specific rehabilitation strategy in achieving successful outcomes after ACL reconstruction.
